# On call

**Published:** 2018-05-31

**Authors:** Michael Natter

**Affiliations:** 1 NYU Langone Medical Center, New York, United States

**Figure:**
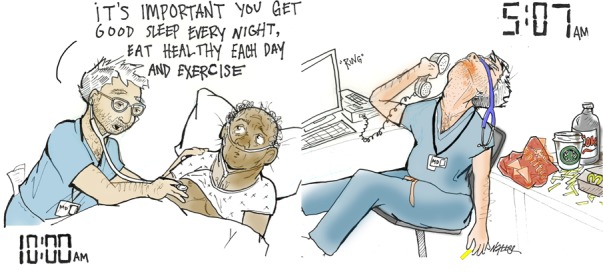


“On Call” playfully reveals a serious irony in medical training: the journey of learning how to make others healthy is wildly unhealthy. Long hours, missed social engagements, poor nutrition, and difficult emotional struggles create an environment seemingly designed to produce burnout. Something must change.

*Michael Natter is a first year internal medicine resident at NYU Langone Medical Center in New York City. He completed medical school at Jefferson Medical College in Philadelphia, PA. While medicine had always been a passion of his, especially since his diagnosis with type 1 diabetes, he decided to study studio art first then came to medicine later in life*.

“On Call” was presented at the White Coat Warm (he)Art exhibit at 2018 Canadian Conference on Medical Education in Halifax, NS.

